# TnseqDiff: identification of conditionally essential genes in transposon sequencing studies

**DOI:** 10.1186/s12859-017-1745-2

**Published:** 2017-07-06

**Authors:** Lili Zhao, Mark T. Anderson, Weisheng Wu, Harry L. T. Mobley, Michael A. Bachman

**Affiliations:** 1 Department of Biostatistics, University of Michigan, 1415 Washington Heights, Ann Arbor, USA; 2 Department of Microbiology and Immunology, School of medicine, University of Michigan, Ann Arbor, USA; 3 BRCF Bioinformatics Core, University of Michigan, Ann Arbor, USA; 4 Department of Pathology, School of medicine, University of Michigan, Ann Arbor, USA

**Keywords:** Transposon sequencing, Essential gene, Differential test, Tn-Seq, InSeq, CD function

## Abstract

**Background:**

Tn-Seq is a high throughput technique for analysis of transposon mutant libraries to determine conditional essentiality of a gene under an experimental condition. A special feature of the Tn-seq data is that multiple mutants in a gene provides independent evidence to prioritize that gene as being essential. The existing methods do not account for this feature or rely on a high-density transposon library. Moreover, these methods are unable to accommodate complex designs.

**Results:**

The method proposed here is specifically designed for the analysis of Tn-Seq data. It utilizes two steps to estimate the conditional essentiality for each gene in the genome. First, it collects evidence of conditional essentiality for each insertion by comparing read counts of that insertion between conditions. Second, it combines insertion-level evidence for the corresponding gene. It deals with data from both low- and high-density transposon libraries and accommodates complex designs. Moreover, it is very fast to implement. The performance of the proposed method was tested on simulated data and experimental Tn-Seq data from *Serratia marcescens* transposon mutant library used to identify genes that contribute to fitness in a murine model of infection.

**Conclusion:**

We describe a new, efficient method for identifying conditionally essential genes in Tn-Seq experiments with high detection sensitivity and specificity. It is implemented as TnseqDiff function in R package Tnseq and can be installed from the Comprehensive R Archive Network, CRAN.

**Electronic supplementary material:**

The online version of this article (doi:10.1186/s12859-017-1745-2) contains supplementary material, which is available to authorized users.

## Background

Large scale transposon mutagenesis coupled with high throughput sequencing (Tn-Seq, also known as INseq, HITS and TraDIS) [1–4] has become a powerful tool to simultaneously assess the essentiality of all genes under experimental conditions. There are mainly two types of data analysis in such experiments: 1) To identify genes required under any growth condition (absolutely essential genes) and 2) to identify conditionally essential genes between conditions (i.e., a differential test). In this paper, we focus on the second analysis. With Tn-Seq, a library of tens of thousands of bacterial mutants is constructed. The location of each insertion mutation and the number of bacteria with that mutation is determined by massively parallel sequencing. By comparing the mutant counts before and after an experimental condition, the fitness contribution (i.e., conditional essentiality) of each gene can be assessed.

To date, analysis of Tn-Seq data has relied on oversimplified *t*-tests or their nonparametric alternatives [5–11]. Recently, several papers considered statistical methods developed for RNA-Seq data [12–14]. These studies applied edgeR [12, 15] to the overdispersed count data to either identify differentially represented (DE) mutants (i.e., the insertion-level inference) [12, 14] or DE genes based on the sum of insertion counts in each gene [13]. For the gene-level inference, however, they ignored special features of the Tn-Seq data. One distinct feature is that each gene is disrupted at multiple locations, where each insertion site represents a unique mutant. When the library is subjected to a selective condition, such as an animal model of infection, each mutant with an insertion in the gene is expected to have decreased abundance in the output samples if that gene is important for fitness. Hence, each insertion site into a particular gene provides independent evidence to prioritize that gene as being conditionally essential in that condition.

Recently the hidden Markov modeling (HMM) has been adapted to identify conditionally essential genes using the insertion-level data [16]. The HMM is a probabilistic statistical model that decodes whether genomic regions belong to a particular biological category given the fold changes in read counts at every insertion site in the genome. A major drawback of the HMM is that it relies on a high-density transposon library to determine whether a gene or region is truly essential (the density is required to be greater than 50%).

Another method that considers the insertion-level data to assess the gene essentiality is the permutation test implemented in software TRANSIT [17]. The permutation test does not require a high-density library, and it identifies essential genes between conditions using a resampling approach. Although the resampling is done on the insertion-level by randomly reshuffling the observed counts at sites in the gene among all the samples, the statistics are based on the total read counts at all the sites for each gene. Additionally, the permutation test has some disadvantages compared to a parametric approach, including 1) a low power with a small number of replicates, 2) misleading results when the samples are correlated or of unequal precision, and 3) inability to accommodate complex design and quality weights [18].

To address all the above limitations, we propose an efficient, parametric method to identify conditionally essential genes based on insertion-level data. The proposed method deals with data from both low- and high-density libraries and is able to accommodate complex designs with multiple inoculum pools and even with multiple conditions. The proposed method was implemented as R package Tnseq (https://CRAN.R-project.org/package=Tnseq).

## Methods

### Data preprocessing

Before applying TnseqDiff, the raw sequence reads need to be processed (e.g., align transposon-flanking sequence reads to genome, filter reads mapped to multiple loci, remove reads from transposons inserted in the 3’ end of a gene that cause loss of function, filtering out spurious insertions by removing insertions with low read counts). The final dataset for analysis contains the read counts of all the insertions in each gene for each sample in the Tn-Seq study. The data processing step can be done using pipelines [13, 17, 19]. The resulting data for analysis is a count matrix, where each column represents a sample from a particular inoculum pool under a specific condition, and each row represents an insertion site in a particular gene in the bacterial genome (see the hypothetical data in Table 1). The default normalization method in TnseqDiff is TMM (trimmed mean of M values) [20]. TnseqDiff also takes the read count data that was already normalized by other methods (see a discussion of normalization methods in [17]).

**Table 1:** Each column represents a sample (S) from the input or output condition. Each row represents an insertion site in a particular gene in the bacterial genome. Each entry is the read counts mapped to a particular insertion site in a particular gene for a particular sample

		Pool I				Pool II					
		Input		Output			Input		Output		
Gene	Location	S1	S2	S1	S2	S3	S1	S2	S1	S2	S3
1	110	478	500	90	100	121	0	0	0	0	0
1	150	810	910	120	10	5	810	910	120	10	5
1	350	910	700	50	80	37	0	0	0	0	0
1	400	1522	1544	142	150	124	1522	1544	142	150	124
1	520	320	240	50	1170	132	320	240	50	1170	132
1000	3110	100	120	20	10	30	210	190	20	0	70

TnseqDiff allows the user to visually evaluate the bias caused by replication process for each sample. Because of asynchronous initiation of DNA replication and cell division, insertions near the origin of replication (ORI) typically are represented as a higher proportion of DNA than insertions farther from the ORI. This is a primary problem when identifying essential genes in a single library and is less of a concern when identifying conditionally essential genes since replication processes are likely to be similar between samples. TnseqDiff provides a method similar to [13] to correct the replication bias when replication processes are different between samples.

TnseqDiff utilizes two steps to estimate the conditional essentiality for each gene in the genome. First, it collects evidence of conditional essentiality for each insertion by comparing read counts of that insertion between conditions. Second, it combines insertion-level evidence to infer the essentiality for the corresponding gene.

### Step 1: collect evidence of conditional essentiality for each insertion

A normal linear modeling is used in TnseqDiff to obtain the insertion-level information. Specifically, log2-counts per million (logcpm) at each insertion site are modelled as a linear function of the condition (i.e., *y*_*ij*_=*α*_*i*_+*β*_*i*_ *x*_*j*_, where *y*_*ij*_ is the logcpm for insertion *i* in sample *j*, and *x*_*j*_ takes 0 if sample *j* is in output and 1 if it is in input). The slope coefficient, *β*_*i*_, in the model represents the log fold-change (logFC), which is the key parameter for the estimation of conditional essentiality. For example, a large logFC (input over output) might indicate stronger evidence for that insertion being conditionally essential. To consider the over-dispersion of the count data, a precision weight is estimated for each observation from the mean-variance relationship of the logcpm and is then entered into the linear modeling [21]. TnseqDiff relies on the Limma package [18, 22] for the above estimation.

To collect evidence of conditional essentiality for each insertion, we construct a confidence distribution (CD) [23–25] for the logFC at each insertion site using estimates from the above linear model. The CD has attracted a surge of attention in recent years. A CD function contains a wealth of information for inferences; much more than a point estimator or a confidence interval. It is a “frequentist" analogue of a Bayesian posterior. Furthermore, it provides a framework to combine evidence through combining CD functions (in our case, combining insertion-level CD functions to make inference for the gene).

The CD function for the *i* ^*th*^ insertion, *H*(*β*_*i*_), is defined as

$$H(\beta_{i})=F_{t_{d_{i}}}\left(\frac{\beta_{i}-\hat{\beta}_{i}}{s_{i}}\right), $$

where $\hat {\beta }_{i}$ is the mean estimate of the logFC, *s*_*i*_ is the standard error and *d*_*i*_ is the degrees of freedom. $F_{t_{d_{i}}}$ is the cumulative distribution function of the $t_{d_{i}}$ distribution. When *β*_*i*_ varies, *H*(*β*_*i*_) forms a function on the parameter space of *β*_*i*_, which contains a wealth of information about the *β*_*i*_, including point estimates (such as mean, median and mode), confidence intervals of various levels and significance testing (see details in [23, 25] and Figure 1 in [25] graphically illustrates the above estimates).

Alternatively we can replace *s*_*i*_ and *d*_*i*_ by the corresponding moderated estimates based on the empirical Bayes method [18, 22]. The CD function constructed based on the moderated estimates is a moderated CD function, which efficiently borrows information from similar insertions to aid inference for any single insertion.

### Step 2: combine insertion-level evidence

TnseqDiff combines the insertion-level CD functions to obtain a single CD function for the corresponding gene. This is accomplished by the use of a simple formula

1 $$\begin{array}{@{}rcl@{}} H_{g}(\beta)=\Phi\left(\frac{1}{\sqrt{\sum_{i=1}^{N} w_{i}^{2}}} \left[w_{1}\Phi^{-1}(u_{1})+\cdots+w_{N} \Phi^{-1}(u_{N})\right]\right), \end{array} $$

where *Φ* is the cumulative distribution function of the standard normal distribution, *u*_*i*_ is the CD function for insertion *i* and *w*_*i*_ (*w*_*i*_≥0) is its weight. If *w*_*i*_=0, insertion *i* is not included in the combined CD function. The combined CD function, *H*_*g*_(*β*), contains essentiality information from all *N* insertions. Here, subscript “*g*" is used to indicate that the combined CD function is on the gene level.

**Fig. 1:**
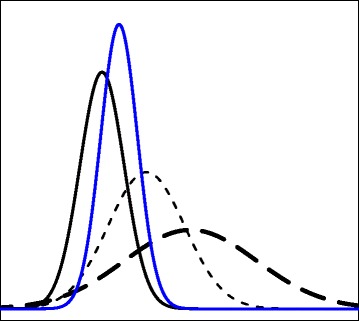
The *black curves* represent insertion-level CD density functions and the *blue curve* is the combined CD density function

It is important to note that the combined CD function, *H*_*g*_(*β*), automatically puts more weight on the insertion-level CD function containing more information even when *w*_*i*_’s are all equal. The idea of combining CD functions is illustrated with a simple example in the Fig. 1. In this figure, three insertion-level CD density functions (black curves) have different means and variances (variances increase from the left to the right curve). The blue curve is the combined CD density function using formula (1) with equal weights. As shown in this figure, the combined function is located near the insertion-level function with less spread (i.e., a smaller variance).

Furthermore, TnseqDiff allows unequal weights for the combination. Insertions with low read counts (≈0) in the input condition might suggest that they are essential for growth in any given condition, therefore, the analysis should exclude or consider a small weight for these insertions. TnseqDiff identifies insertions with “low" counts using a fast dynamic programming algorithm for optimal univariate 2-means clustering [26]. The insertions that are clustered into the group with a smaller mean are assigned weights less than one, specifically, these weights are estimated from an exponential function (the smallest count gets a weight close to zero, while the largest count gets a weight close to one). We call this weight function hc. TnseqDiff also takes weights specified by the user. For example, the probability of an insertion being absolutely essential can be used as the weight for that insertion and obtained from a separate method (such as the method in [16] or [27]).

### Identify conditionally essential genes based on the combined CD function

TnseqDiff estimates the conditional essentiality for a particular gene using the combined CD function *H*_*g*_(*β*). As shown in [25], the median logFC is estimated based on $H_{g}^{-1}(\frac {1}{2})$. Specifically, TnseqDiff uses a numeric algorithm to solve for *β*_*g*_ in the equation

$$ \sum_{i=1}^{N} w_{i} \Phi^{-1}\left(F_{t_{d_{i}}}\left(\frac{\beta_{g}-\hat{\beta}_{i}}{s_{i}}\right)\right)=0 $$

In a simple case where *w*_1_=⋯=*w*_*N*_≡1 and the *t* distribution can be approximated by a normal distribution, the median logFC is simplified as

$$\text{Median logFC}= \frac{\sum_{i}^{N} \widehat{\beta}_{i}/s_{i}}{\sum_{i}^{N} 1/s_{i}} $$

In this case, the median logFC is a weighted average of the insertion-level logFC estimates, with the weight inversely proportional to the standard error.

Similarly, the lower and upper bound of a level 100(1−*a*)% confidence interval can be calculated by solving equation

$$\sum_{i=1}^{N} w_{i} \Phi^{-1}(F_{t_{d_{i}}}((\beta_{g}-\hat{\beta}_{i})/s_{i}))- \left(\sum_{i}^{N} w_{i} \right)^{\frac{1}{2}} \Phi^{-1}(a/2)=0 $$

and

$$\sum_{i=1}^{N} w_{i}\Phi^{-1}\left(F_{t_{d_{i}}}\left(\left(\beta_{g}\,-\,\hat{\beta}_{i}\right)/s_{i}\right)\right)\,-\, \left(\sum_{i}^{N} w_{i} \right)^{\frac{1}{2}}\Phi^{-1}(1-a/2)\,=\,0, $$

respectively.

For testing if a gene is conditionally non-essential versus essential, the hypotheses are *H*_0_:*β*_*g*_≤0 vs. *H*_1_:*β*_*g*_>0. As defined in [25], the one-sided *p*-value is simply *H*_*g*_(0), where

$$H_{g}(0)= \Phi \left(\frac{1}{\sqrt{\sum_{i}^{N} w_{i}}} \sum_{i=1}^{N} w_{i} \Phi^{-1} \left(F_{t_{d_{i}}}\left(-\frac{\hat{\beta}_{i}}{s_{i}} \right)\right)\right). $$

The two-sided *p*-value is 2× min{*H*_*g*_(0),1−*H*_*g*_(0)} (TnseqDiff provides a two-sided *p*-value). These *p*-values are then adjusted for multiple testing using the Benjamini-Hochberg Procedure [28].

In real applications, differentially represented genes are generally selected based on both the adjusted *p*-value and the fold-change (FC). Tnseqdiff uses the median logFC as defined above, that is, FC =2^median logFC^. It is important to note that TnseqDiff calculates the *p*-value and median logFC from the combined CD function. If only interested in identifying conditionally essential genes (i.e., identifying genes with decreased counts in output), we can set the rule as the FC (input over output) ≥2 and the adjusted *p*-value <0.025 in a two-sided test (or *p*-value <0.05 in a one-sided test).

In addition to the above estimates, TnseqDiff also provides descriptive statistics for each gene, including the number of (unique) insertions in input samples and averaged counts in input and output samples (after accounting for the differences in library sizes).

Our proposed method is much simpler to implement than a model-based approach and it can be easily extended to analyze more complex designs.

### Analyze designs with multiple inoculum pools

Mutant pools are often too large (∼ 50,000 random mutants for a 5 Mbp gemome) to be tested in one mouse, or an experimental “bottleneck” would cause random loss of mutants from a large inoculum. In these cases, the mutant library is split and smaller pools are used to inoculate separate sets of mice. Hence, different mutants within a particular gene are tested in different mice. It would be inaccurate to sum over the insertion counts that are observed in different mice due to the loss of biological variability. However, our method is directly applicable to such designs since samples at each insertion site in different pools are independent (the only requirement for combining CD functions). TnseqDiff first combines insertion-level CD functions to obtain a CD function for each gene in a given pool, and then it combines CD functions from multiple pools for each gene to obtain a single CD function for identifying conditionally essential genes.

## Results and discussion

### Simulation studies

We ran simulation studies to investigate our proposed methods and compared them to 1) the permutation test in the TRANSIT software [17] and 2) the negative binomial test in the ESSENTIALS software [13]. In the permutation test, the read counts at all the sites and all samples in each condition are summed for each gene. The difference in the sum between conditions was calculated. The significance of this difference was evaluated by comparing to a resampling distribution generated from randomly reshuffling the observed counts at sites in the gene among all the samples. A *p*-value was then derived from the proportion of 10,000 reshuffled samples that have a difference more extreme than that observed in the actual experimental data. ESSENTIALS used the method in edgeR to identify DE genes based on the total gene counts, therefore, we directly applied edgeR to the datasets after obtaining the total gene counts by summing over the insertion counts for each gene.

To make simulation studies more realistic, the data and insertion distributions in simulated datsets were similar to a real dataset. The real dataset was generated from a *Serratia marcescens* transposon mutant library with the objective of identifying bacterial genes that contribute to fitness in a murine model of bloodstream infection [29] (details are shown in the next section). It consists of five inoculum pools with 2 input and 4 output samples per pool. We merged data from five pools and assumed that insertions at the same genomic location in different pools were different insertions. After data normalization, we averaged the two input samples and excluded insertions with an averaged count < 5 (remaining insertions were considered as true insertions). The final dataset consists of 4,075 genes with 42,639 insertions. The number of insertions per gene ranged from 1 to 202 (median is 8, the first and third quartile is 4 and 14, respectively). This insertion distribution was assumed in the first two simulation studies. Input data were generated from Poisson distributions because input samples (in vitro) are technical replicates, while output data were generated from negative binomial (NB) distributions because the output samples (in vivo) are biological replicates.

#### The first simulation study: all insertions are genuine insertions

In this study, we focused on identifying conditionally essential genes based on true insertion data and assumed that absolutely essential genes and spurious insertions (in vitro) have been removed. Given the insertion distribution in the real dataset, we first generated the input data for each insertion from a Poisson distribution with the mean parameter equal to the averaged count. Then we randomly selected 10% of the genes to be under-represented (i.e., conditionally essential) and 5% to be over-represented in the output samples. For insertions in under-represented genes, logFCs were generated from a left truncated standard normal distribution, while insertions in over-represented genes were generated from a right truncated standard normal distribution. For non-DE genes, logFCs were fixed to be zero. Finally, we generated the output data from a NB distribution with the mean equal to the product of the input mean and the FC. Rather than fixing the dispersion parameter to be the same for all insertions, we generated dispersion parameters from a gamma distribution with a shape =1, scale =0.5 (these two parameters were determined based on the real dataset). In this study, we tried two sample sizes: 1) 2 input vs 4 output samples, and 2) 1 input sample vs 3 output samples.

We applied TnseqDiff to 20 simulated datasets as described above and assumed equal weights for combining the insertion-level CD functions. We considered both moderated and unmoderated CD functions in TnseqDiff and call them moderated and unmoderated TnseqDiff.

**Fig. 2:**
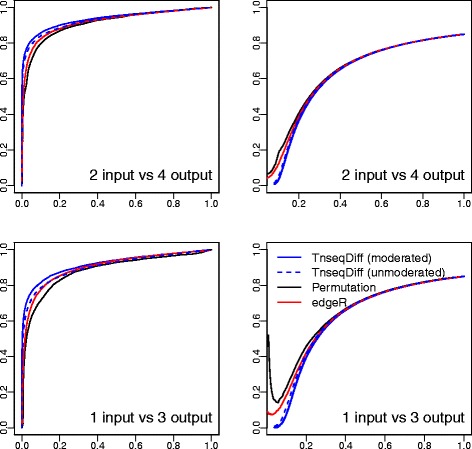
ROC curves (*left*) where *y* axis is the true positive rate and *x* axis is the false positive rate and False discovery curves (*right*) where *y* axis is the percentage false discoveries and *x* axis is the percentage of selected genes. Four methods applied to 20 simulated datasets and results are summarized over all datasets

*Simulation results:* As shown in Fig. 2, TnseqDiff performed significantly better than edgeR and the permutation test under the two studied sample sizes, as evidenced by improved accuracy in separating the truly DE and non-DE genes and a much smaller false discovery rate given the same number of selected genes. Moreover, moderated TnseqDiff performed slightly better than the unmoderated TnseqDiff. Similar conclusions can be reached for the conditionally essential gene detection (i.e., the one-sided test) except that the unmoderated TnseqDiff is similar to the moderated TnseqDiff (ROC and False discovery curves were shown in Additional file 1).

#### The second simulation study: some insertions are spurious insertions

In this study, we included 500 (about 10% of bacterial genome) absolutely essential genes in each simulated dataset. Since an absolutely essential gene should not contain any real insertion, we generated low read count data for these spurious insertions from a Poisson distribution with rate =3 (1-14 “insertions" were assumed within each absolutely essential gene). Additionally, 2,132 spurious insertions (5% of the total 42,639 insertions) were randomly added to the bacterial genome such that a DE gene may contain false insertions. The rest of the simulations were the same as in the first simulation study. This study has 2 input vs 4 output samples.

We applied moderated TnseqDiff to 20 simulated datasets as described above and considered the equal and the hc weight function. The hc weight function downweighs spurious insertions in the analysis (see details in step 2 of the Method section).

**Fig. 3:**
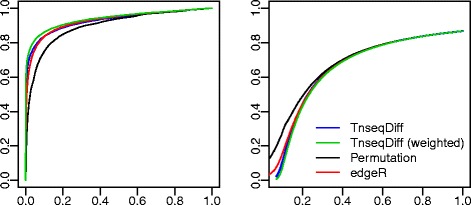
ROC curves (*left*) where *y* axis is the true positive rate and *x* axis is the false positive rate and False discovery curves (*right*) where *y* axis is the percentage false discoveries and *x* axis is the percentage of selected genes. TnseqDiff assumed equal weights and TnseqDiff (weighted) used the hc weight function. Four methods applied to 20 simulated datasets and results are summarized over all datasets

*Simulation results:* As shown in Fig. 3, TnseqDiff with equal weights performed similarly, or slightly better in terms of the false discovery rate, than edgeR and the permutation test. The TnseqDiff with the hc weight function performed better than the TnseqDiff with the equal weight function. Furthermore, we found that all absolutely essential genes were correctly identified as non-DE genes in the weighted TnseqDiff and edgeR, while 68 (13.6%) absolutely essential genes were wrongly identified as DE genes in the permutation test.

#### The third simulation study: each gene has a fixed number of insertions

To investigate the effect of number of insertions per gene on the model performance, we assumed that each gene has a fixed number of insertions (denoted by *n*). Each simulated dataset consists of 5000 genes with *n*=1,3,5,10,20,30, or 50. We first sampled 5000 genes containing at least *n* sites from the above 4075 genes with replacement (the sampling weight for each gene is proportional of the number of sites in that gene). Then we sampled *n* mean parameters from each gene with replacement and these parameters were used in the Poisson distribution to generate the input data. The rest of the simulations are the same as in the first simulation study. This study has 2 input vs 4 output samples.

We applied both the moderated and unmoderated TnseqDiff to 10 simulated datasets as described above. Since all insertions are true insertions, we assumed equal weight in TnseqDiff.

**Fig. 4:**
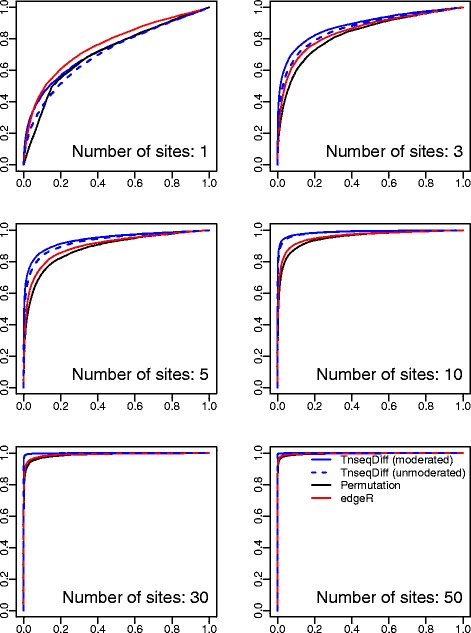
ROC curves where *y* axis is the true positive rate and *x* axis is the false positive rate. Four methods applied to datasets consisting of 2 input and 4 output samples. Each plot presents a scenario for a fixed number of sites per gene

**Fig. 5:**
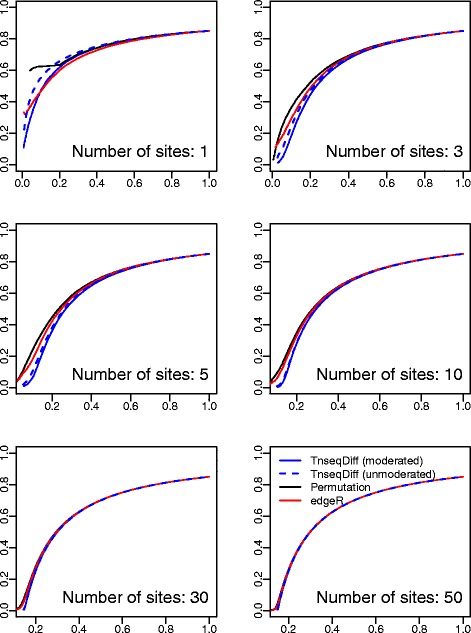
False discovery curves where *y* axis is the percentage false discoveries and *x* axis is the percentage of selected genes. Four methods applied to datasets consisting of 2 input and 4 output samples. Each plot presents a scenario for a fixed number of sites per gene

*Simulation results:* As shown in Figs. 4 and 5, TnseqDiff performed significantly better than edgeR and the permutation test when the number of insertions is >1. When there is just one insertion per gene, TnseqDiff is equivalent to Limma for detecting DE genes (no CD function combining in this case), and the moderated TnseqDiff performed better than the unmoderated TnseqDiff since the moderated estimates borrowed information from similar insertions across all genes. Furthermore, all methods had increased accuracy when the number of insertions per gene was increased. In other words, a gene with a larger number of insertions contains more information and is more likely to be identified as a DE or non-DE gene correctly.

To our surprise, the permutation test performed the worst in all studied scenarios. This could be due to the fact that the permutation test requires that the two distributions are identical [30], however, Tn-Seq studies generally have very different distributions for the input and output data.

**Fig. 6:**
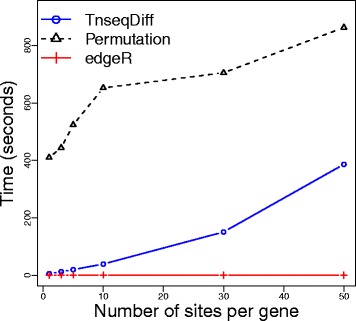
Computation time for three methods applied to a dataset with 2 input and 4 output samples and 5000 genes per sample. TnseqDiff used moderated estimates. There methods were run on a quad-core Intel Xeon 2.10 GHz 8 GB RAM x64 computer

Furthermore, TnseqDiff is much faster to implement than the permutation test especially when the number of insertion sites per gene is small (see Fig. 6).

### Application to a real transposon dataset

We applied TnseqDiff to a published Tn-Seq dataset [29]. The Tn-Seq dataset was generated from a *Serratia marcescens* transposon mutant library with the objective of identifying bacterial genes that contribute to fitness in a murine model of bloodstream infection. A mariner-based transposon encoded in suicide plasmid pSAM-Cm [1] was used to generate a random library of transposon insertion mutants in strain UMH9. An initial mutant library of >32,000 unique transposon insertion mutants was equally split into five inoculum pools. Each pool was used to infect 4 mice and spleens from infected mice were collected after 24 hrs. The insertion sites from input and output pools were PCR-amplified and then sequenced via the Illumina HiSeq platform using 50 cycle single-end reads [31]. Sequence reads were mapped to the UMH9 annotated genome using the ESSENTIALS pipeline with default parameter settings. One output sample from each of pools 3-5 was eliminated from the analysis due to mice that succumbed to infection or insufficient PCR product for sequencing. The final dataset consisted of 4106 genes with at least one transposon insertion, and the number of insertions for a given gene ranged from 1 to 322, with over 50% of the genes having 12 or less insertions. HMM approach is not appropriate for analyzing this dataset since the density of the transposon library is not high.

**Fig. 7:**
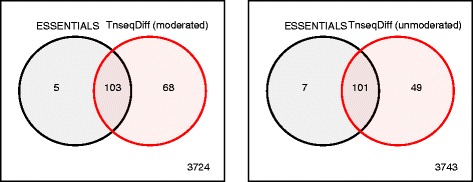
Overlap of conditionally essential genes from ESSENTIALS and TnseqDiff. A gene is essential if the fold-change (input over output) ≥2 and the adjusted *p*-value <0.025

**Fig. 8:**
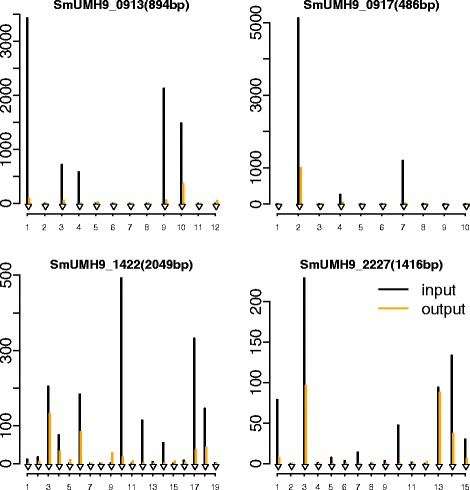
Distribution of insertion counts in four genes. The x-axis is the location and each insertion site is indicated by a black arrowhead. The y-axis is the averaged normalized read counts for input (*black*) and output (*orange*) samples

In TnseqDiff (moderated or unmoderated), equal weights were assumed because the data has been pre-processed using the ESSENTIALS to exclude absolutely essential gene detection. Conditionally essential genes were determined based on the fold-change (input over output) ≥2 and the adjusted *p*-value <0.025. We also applied ESSENTIALS to the same dataset. As shown in Fig. 7, majority of fitness genes were identified by both TnseqDiff and ESSENTIALS and moderated TnseqDiff identified 21 more genes than the unmoderated TnseqDiff. Seven of these genes, encoding a wide range of biological functions and identified by TnseqDiff (moderated and unmoderated) and ESSENTIALS, were chosen for validation of the Tn-Seq screen. Deletion-insertion mutations were constructed for each of the genes and the resulting strains were tested for in vivo fitness defects in competition with the wild-type strain using the murine bacteremia model. The results from these experiments confirmed that six of the seven tested genes contribute to *S. marcescens* fitness in the mammalian host. Importantly, none of the seven mutants exhibited a general growth defect when cultured in vitro. Figure 8 shows four genes that were identified as conditionally essential by TnseqDiff but not by ESSENTIALS. Genes SmUMH9_0913 (*galF*) and SmUMH9_0917 (*neuA*) are both located in the 18-gene *S. marcescens* capsule biosynthesis locus, within which other genes are important for fitness [29]. Genes SmUMH9_1422 and SmUMH9_2227 are predicted to be co-transcribed with a functionally-related adjacent gene that was identified by both TnseqDiff and ESSENTIALS. Complete analysis results from ESSENTIALS and TnseqDiff were presented in Additional file 2.

## Conclusions

We developed methods that are specifically designed for analyzing Tn-Seq data and implemented these methods in the TnseqDiff function in R package Tnseq. TnseqDiff takes into account the unique features of Tn-Seq data and identifies conditionally essential genes using insertion-level data. TnseqDiff handles data from both low- and high-density transposon libraries. We have demonstrated its advantages over the existing methods, including 1) better performance in separating true DE and non-DE genes and a smaller false discovery rate, 2) a much faster computation time, and 3) the ability to accommodate complex designs (for example, designs with multiple pools). TnseqDiff can be easily extended to analyze data with multiple experimental conditions. In this case, data from all conditions will be included in the linear model, and coefficient estimates or estimates of interested contrasts can be used to construct the CD function for testing interested hypotheses.

It is worth noting that, unlike the HMM method, TnseqDiff does not rely on a high-density transposon library for inference. It focuses on identifying conditionally essential genes and is most efficient when absolutely essential genes and spurious insertions have been removed first. TnseqDiff with the hc weight function downweighed spurious insertions and it worked well in simulation studies where absolutely essential genes and spurious insertions were present in the bacterial genome. These weights can also be obtained using other existing softwares for the absolutely essential gene detection (such as ARTIST or TRANSIT). In these softwares, an estimated probability for an insertion to be absolutely essential can be considered as the weight for that insertion and incorporated into TnseqDiff for the differential test.

Unlike the HMM approach in ARTIST, TnseqDiff is annotation-dependent. It evaluates conditional essentiality for previously-annotated genomic features (e.g., ORFs, ncRNAs). However, TnseqDiff allows inference for intergenic regions and subdomains of ORFs if these regions are pre-defined in the dataset by combining the insertions within that region for inference.

## Additional files

Additional file 1 ROC and False discovery curves for conditional essential gene detection in the first simulation study. (PDF 128 kb)

Additional file 2 Analysis results from ESSENTIALS and TnseqDiff for the *S. marcescens* study. (XLSX 1040 kb)

## Abbreviations

CD: Confidence distribution
DE: Differentially represented
FC: Fold change
HMM: Hidden Markov modeling
NB: Negative binomial
ORI: Origin of replication
TMM: Trimmed mean of M values
Tn-Seq: Experiments with large scale transposon mutagenesis coupled with high throughput sequencing
