# Supplementary material for: TnseqDiff: identification of conditionally essential genes in transposon sequencing studies
Source: BMC Bioinformatics. 2017 Jul 6;18:326. doi: 10.1186/s12859-017-1745-2 (PMC5500955; doi:10.1186/s12859-017-1745-2)
Supplement: Supplementary file 1 — ROC and False discovery curves for conditional essential gene detection in the first simulation study. (PDF 128 kb) [file 12859_2017_1745_MOESM1_ESM.pdf]

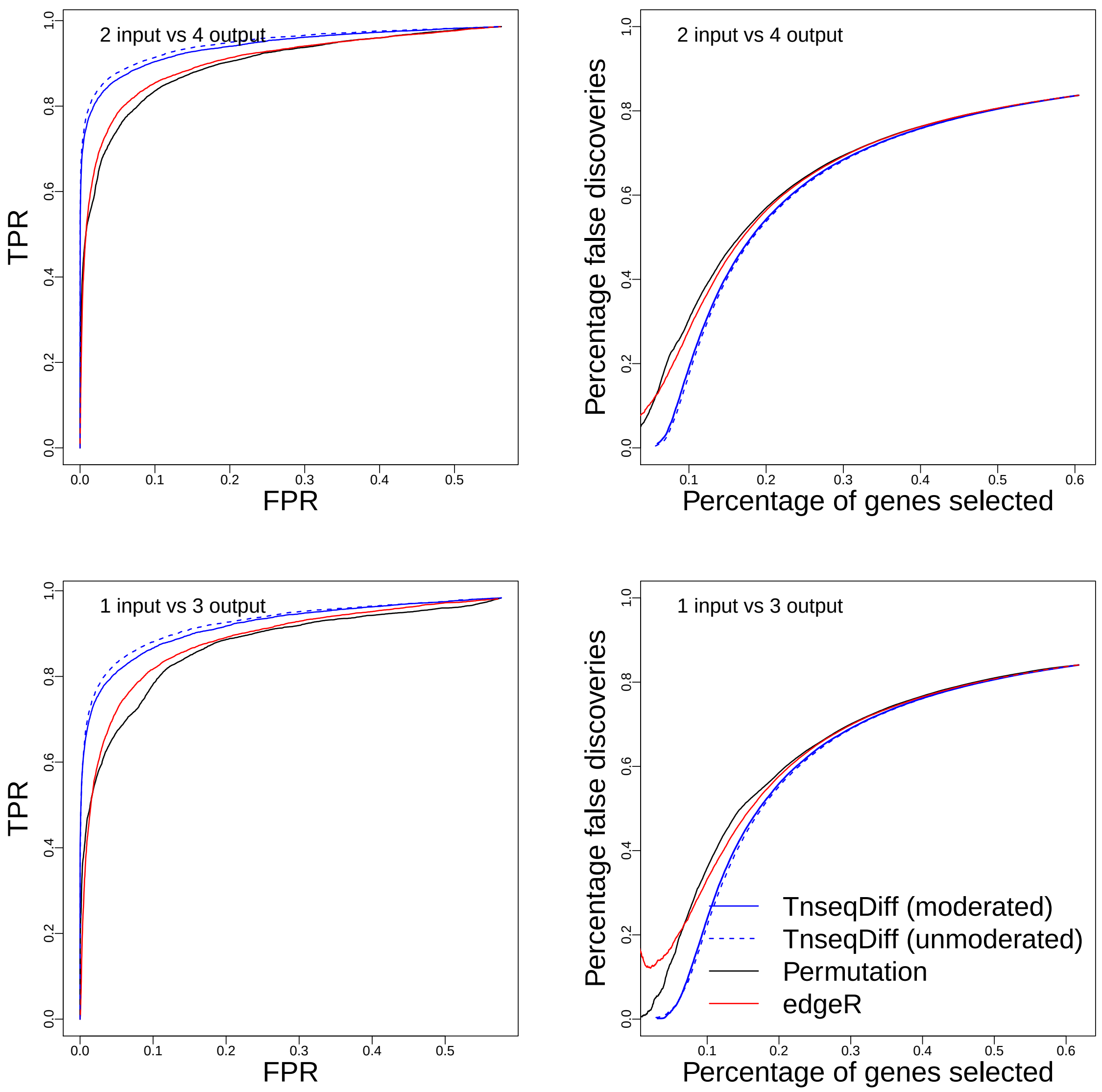

Figure1S: Eevaluate the model performance for identifying conditionally essential genes. ROC curves (left) where y axis is the true positive rate and x axis is the false positive rate and False discovery curves (right) where y axis is the percentage false discoveries and x axis is the percentage of selected genes. Four methods applied to 20 simulated datasets and results are summarized over all datasets.
